# Evaluation of Trans-Anal Endorectal Pull-Through Outcomes in Hirschsprung’s Disease in Different Age Groups: A Comprehensive Systematic Review

**DOI:** 10.34172/aim.28183

**Published:** 2024-07-01

**Authors:** Farshid Ghasemi Meidansar, Mohammad Moradi, Seyed Ali Nabipoorashrafi, Seyyed Javad Nasiri, Tahereh Chavoshi, Mohammad Aldraji, Fariba Jahangiri

**Affiliations:** ^1^Department of General Surgery, School of Medicine, Iran University of Medical Sciences, Tehran, Iran; ^2^Department of Pediatric Surgery, Ali-Asghar Children Hospital, Iran University of Medical Sciences, Tehran, Iran; ^3^Department of Anesthesiology, School of Medicine, Iran University of Medical Sciences, Tehran, Iran; ^4^School of Medicine, Shahed University, Tehran, Iran

**Keywords:** Hirschsprung’s disease, Infant, Neonate, Systematic review, Trans-anal pull through

## Abstract

**Background:**

The timing of trans-anal endorectal pull-through (TAEPT) for Hirschsprung’s disease (HD) is controversial. Early endorectal pull-through avoids the occurrence of preoperative enterocolitis. However, delayed pull-through (≥31 days) enables postnatal maturation of the anal canal and sphincter complex. The aim of this study was to identify the best age to perform trans-anal pull-through according to the literature.

**Methods:**

This is a comprehensive systematic review. All articles published from 2010 to 2022 were searched in the Web of Science, Ovid Medline, PubMed, CINAHIL, and Embase databases, using the keywords HD, delayed or early treatment, trans-anal pull-through surgery, age, sex or gender, complications and outcomes. Articles that met the inclusion criteria with good to fair quality according to the Newcastle-Ottawa quality assessment and low bias score in the Cochran collaboration tool were reviewed.

**Results:**

Sixteen studies were eligible to be reviewed. The overall results of this study showed that due to more common short-term complications at neonatal period and lower contrast enema diagnostic accuracy in determining the transition zone, it seems to be reasonable decision to postpone surgery until the child is several months old. There was also no difference in terms of complications and outcomes of trans-anal pull-through surgery between females and males.

**Conclusion:**

It is not recommended to delay surgery too much for ages over 1 year. Ages between 3 and 12 months can be a good time for interventional treatment for HD.

## Introduction

 Hirschsprung’s disease (HD) is a pretty common surgical disease in children studied by pediatric surgeons and researchers. The prevalence of Hirschsprung is 1 per 5000 live births and the probability of transmission to the next generation is about 3%.^1,2^ This congenital disease is caused by a developmental disorder of the intestinal nervous system and is characterized by absence of ganglion cells in the submucosal layer (Meissner) and the myenteric network of the distal colon. The ganglion-free segment in the intestine lacks normal movement, so the proximal intestine dilates, leading to functional bowel obstruction, putting these patients at high risk for enterocolitis.^3^

 Most cases of HD are now diagnosed in infancy. HD should be considered in neonates who do not have meconium excretion within 48 hours of birth or have vomiting and abdominal distention. Nonetheless, full-thickness biopsy of the rectal wall has been suggested as the most reliable test to endorse the diagnosis.^4^ Normal relaxation of the internal sphincter in response to rectal dilation is also impaired, which forms the basis of manometric diagnostic modality.^5^

 Patients who have a confirmed diagnosis of HD undergo corrective surgery. In 1948, Swenson and Bill performed the first corrective surgery to remove the aganglionic segment of the colon followed by coloanal anastomosis.^6,7^ Traditionally, treatment involves a diverting colostomy at the time of diagnosis, and then, when the child grows older and weighs more than 10 kg, a definitive repair is considered. In 1998, De la Torre-Mondragón et al developed a single stage trans-anal pull-through for HD.^8^

 In patients who respond to enema and rectal lavage, Botox can be used temporarily until the final pull-through operation is performed.^9^ In recent years, pull-through from the anal canal (TAEPT) has received much attention. In this method, laparotomy is not always required and the rectum is usually removed by maintaining the surrounding muscle cuff, leading to less damage to adjacent tissues and nerves.^10,11^

 The advantages of TAEPT include easy technique, no need for colostomy, low bleeding rate and short hospital stay compared to other modalities. Nevertheless, there is no definite recommendation for the best age to perform the operation. Therefore, in this study, we investigated the best age group who are suitable to undergo corrective surgery with the fewest complications and best outcomes. Besides, the operation outcomes in males and females were compared.

## Methods and Materials

 This was a comprehensive systematic review. The present study included all studies regarding trans-anal pull-through in patients with HD. We included all studies evaluating this method in children with different age groups from 2010 to 2022. Non-English articles were excluded. Case reports, case series and expert opinions were also excluded. If necessary, the authors were contacted to provide more information. Moreover, we removed studies that did not report information regarding response to trans-anal pull-through surgery and its complications in Hirschsprung patients or did not have sufficient data. Five main databases were searched, including Web of Science, Ovid Medline, PubMed, CINAHIL, and Embase. The Google Scholar database was searched finally to ensure that the systematic search was complete. Abstracts published in conferences or dissertations on trans-anal pull-through in patients with Hirschsprung’s were also considered as much as possible. Studies that included patients with concurrent congenital abnormalities were excluded.

 The main keywords included Hirschsprung’s, HD, congenital megacolon, trans-anal, pull-through, aganglionosis, aganglionic segment, aganglionic bowel, rectosigmoid colon, anorectal stenosis, enterocolitis, soiling, fecal continency, bowel continency, fecal soiling, pediatric, newborn, child, infant, infancy, neonate and Bowel obstruction.

###  Bias Assessment Tool

 The bias of included studies was checked by two independent authors. In case of disagreement between the two authors, a consensus was reached through discussion and exchange of views or by requesting a third opinion. The Joanna Briggs Institute Critical Appraisal tool^12^ was used to assess the eligibility of studies. This tool consists of 10 questions in three main sections of design, conduct and analysis. Each question scores yes, no, unclear or not applicable. For instance, question No. 1 is “Was the sample representative of the target population?”^12^.

 Data were extracted according to a standard protocol. The extracted information included study design, year of publication, authors’ name, sample size, surgical complications including enterocolitis, anastomotic stenosis, fecal continence and constipation, age and sex.

## Results

**Figure 1:**
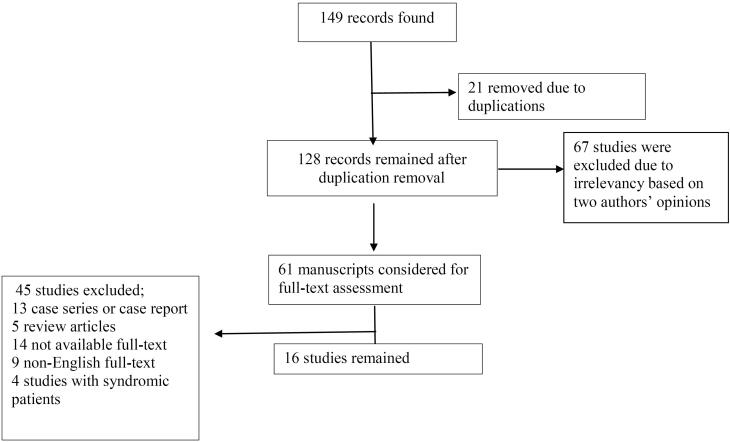
The Study Flow Chart

 Initially, 149 studies were found. After the primary review, duplicate studies (21 records) were excluded. After evaluating these 128 studies by their titles and abstracts, 61 articles were considered for full-text evaluation. Of these 61 studies, 45 studies were omitted for various reasons depicted in Figure 1, and 16 studies remained for the final analysis. The study flowchart is depicted in Figure 1. Our results were classified into two main categories including the results of trans-anal pull-through surgery based on age and gender.

###  Age 

 A large retrospective cohort study by Lu et al in both neonatal and non-neonatal groups (650 patients) found that a single-stage trans-anal pull-through in the non-neonatal period may be more appropriate than the neonatal period. There was a higher rate of perianal excoriation, anastomotic stenosis and leakage, postoperative enterocolitis, and postoperative incontinence in neonates compared to non-neonates.^13^ Furthermore, in another study by the same authors, Lu et al recommended home rectal irrigation, followed by a delayed and planned surgery, as intervention in the neonatal period lacks enough diagnostic accuracy and a higher rate of post-op enterocolitis.^14^ Moreover, in a retrospective cohort study in 2019, Zhu et al stated that children with Hirschsprung’s under 3 months of age had lower rates of accurate diagnostic results and poorer postoperative outcomes. They suggested that it may be more appropriate to wait until the child is above three months to perform surgery.^15^

 A study by Freedman-Weiss et al on 282 patients found that for appropriately selected patients with HD, delaying pull-through until the second month of life is associated with lower total and postoperative stays without increased re-admissions or complications.^16^ However, in another study by Beltman et al, multivariate analysis showed that older age at surgery (median 105 days) increases the risk of developing postoperative complications (OR = 1.00, 95% CI = 1.00‒1.01, *P* = 0.041).^17^

 On the other hand, Zakaria’s study compared two groups of patients regarding outcome and complications. The mean age in groups A and B was 14.02 ± 10.3 and 69.9 ± 32 months, respectively. They suggested that it may be better to perform surgery at a younger age and delaying surgery is associated with comorbidities. Most children at younger age showed no abnormal defecation problems, and had an excellent fecal continence score rate.^18^ Besides, in another study by the same author, fecal incontinence was less frequent when the operation was performed between 6 months to 2 years of age.^19^ Moreover, in Khalil’s study, higher age at surgery significantly affected physical function (β = -0.686, *P* = 0.001) and reduced school performance (β = -0.2279, *P* = 0.027). They indicated that age at surgery (patients’ age was 7-19 months) had a significant negative correlation with quality of life, as children who underwent surgery at a younger age had better quality of life.^20^

 In another study by Kumar et al, the implications of the single-stage trans-anal endorectal pull-through (TAEPT) were assessed retrospectively. Perianal excoriation was reported in 60% of patients, more commonly in neonates. Besides, stool frequency was reported to be more than 5 to 7 per day in all neonates early after the procedure. Moreover, blood transfusion was higher in children above one year. They concluded that delaying surgery is not logical and neonates and infants might benefit the most compared to other age groups ^21^. Besides, Kastenberg et al compared delayed primary endorectal pull-through ( ≥ 31 days). The median age at operation was 98 days (IQR 61-188 days) for infants. They assess 82 patients, 49 neonates and 33 non-neonates. Fifteen neonates compared to five non-neonates developed fecal incontinence (*P* value = 0.13). Besides, enterocolitis and other complications were not different between the two groups.^22^

 Furthermore, in a retrospective study by Dahal et al on 113 children, younger age at surgery (under 3 years) was associated with lower bowel frequency (less than 3 times a day) (*P* < 0.05). There was a significantly higher frequency of stool in patients aged more than 36 months and those with a resected colon more than 30 cm.^23^

 On the other hand, a study by Miyano et al showed that age at surgery was not correlated with postoperative bowel function in HD.^24^ They reported that only operation duration was significantly higher in patients older than 4 years, and other outcomes did not differ significantly. Furthermore, in a study by Hoff et al, there was no risk factor for short-term complication using the Clavien-Dindo grading system, including age at surgery [median age 62 days] (OR = 2.97, 95% CI = 9.93‒0.92).^25^

 Nonetheless, in a study by Byström et al, children who underwent TAEPT for Hirschsprung had significantly impaired bowel function scores compared with healthy controls in several aspects. There were no differences between age groups in this study [median age at TAEPT was 57 days (12‒3,355)], indicating impaired bowel function after TAEPT.^26^

 On the other hand, in a multicenter study in Scandinavia to evaluate the predictors of functional outcomes, age at surgery did not have a significant effect on poor outcomes using a multivariate model.^27^ This study was a retrospective investigation mainly to identify long-term complications of children with HDs after operation. In addition, in a very recent study by Zhang et al, 229 neonates who underwent TAEPT were reviewed. They reported that operation in the neonatal period was quite safe with few complications (age of 6‒28 days).^28^ In 62 patients, there was no radiological transition zone (27.1%). Early post-op complications (wound infection, dehiscence, sepsis, etc) occurred in 26 patients (11.4%). Enterocolitis was noted in 16 (7%). The follow-up period ranged from 1.2 years to 14 years. Delayed complications (stricture, fistula, prolapse) were reported in 6 patients. Soiling persisted in 22 patients (20.8%).

###  Gender 

 Dahal et al reported the same incidence of postoperative complications in both males and females. There were no statistically significant differences in terms of stool frequency less than 3 times a day (male/female; 70%/100%, *P* = 0.09), soiling (male/female; 7%/12.5%, *P* = 0.4) and constipation (male/female; 3%/0%) ^23^. In another study, the prevalence of social problems after surgery was not affected by gender (*P* < 0.05).^29^ In addition, Dehghan et al compared the two methods of trans-abdominal or trans-anal pull-through in children with Hirschsprung, and reported no significant differences regarding complications in males and females.^30^ In another study, sphincter function was not related to the patient’s gender.^31^

 In two different studies, there were no significant differences between males and females during the first 30 days after surgery in terms of Clavien-Dindo grading, anastomotic stenosis, postoperative enterocolitis, bleeding and wound infection, length of hospital stay after surgery, re-admission within 30 days after surgery, and the need for re-operation.^25,26^

 In the study by Byström et al,^26^ the comparison of bowel function score between males and females with HD showed no significant difference. In another study ^17^, in univariate analysis, gender was not reported as a risk factor for postoperative complications (OR = 1.27, 95% CI = 0.38‒4.23, *P* = 0.698). In the study by Gunadi et al,^32^ no association between gender and voluntary bowel movement (VBM) was observed in Hirschsprung patients after TAEPT. A summary of main studies used in this systematic review and their outcomes is depicted in Table 1.

**Table 1:** Summary of Studies Used in This Systematic Review

**Author(s)**	**Publish Year**	**Subjects**	**Male**	**Female**	**Study Design**	**Quality**	**Outcome**
Lu et al^13^	2017	650 children in two groups of neonates and non-neonates	497	153	Retrospective cohort	Good	TAEPT in the non-neonatal period may be more appropriate than in the neonatal period, especially regarding post-op complications.
Zhu et al^15^	2019	62 infants < 3 months and 136 infants aged 3-12 months	158	40	Retrospective cohort	Good	Infants ≤ 3 months old with Hirschsprung's disease showed lower rates of accurate and conclusive diagnostic results and more postoperative complications.
Freedman-Weiss et al ^16^	2019	282 patients in two groups of < 31 days and 31-120 days old	231	51	Retrospective cohort	Good	Delaying pull-through until the second month of life is associated with lower total and postoperative stays without increased readmissions or complications.
Beltman et al^17^	2021	106 patients underwent TAEPT (Median age at time of surgery:105 days)	80	26	Retrospective	Good	Older age at time of surgery was a risk factor for postoperative complications.
Zakaria ^18^	2012	40 patients in two age groups (6-42 months and 3.5-13 years old)	28	12	Comparative retrospective cohort	Good	Group A had fewer defecation problems, and had excellent fecal continence scores.
Zakaria et al ^19^	2012	50 patients in two age groups	27	23	Retrospective cohort	Good	The earlier the surgery of HD, the lower the incidence of fecal incontinence (6 months - 2 years had better results than > 2 years).
Khalil et al ^20^	2015	70 patients	37	16	Retrospective	Fair	Surgery at a younger age (patient age 7-19 months) is associated with better quality of life.
Kumar et al ^21^	2019	30 patients including 10 neonates, 13 infants and 7 children	26	4	Retrospective/Prospective cohort	Good	Excoriation was higher in neonates, but overall outcomes were better in neonates.
Kastenberg et al ^22^	2021	82 patients	68	14	Retrospective	Good	Fecal incontinence more frequent in neonates (*P* value = 0.13), but overall equivalent outcomes.
Dahal et al ^23^	2011	131 children with HD aged 7 days to 14 years	112	19	Retrospective	Good	Younger age at surgery (under 3 years) was associated with lower bowel frequency (less than 3 times a day).
Miyano et al ^24^	2017	106 patients underwent laparoscopic pull-through in 4 age groups ( < 3 months, 3-11 months, 1-3 years and > 3 years)	68	38	Prospective cohort	Good	Age at surgery was not correlated with postoperative bowel function in Hirschsprung's disease.
Hoff et al ^25^	2019	69 patients	51	18	Cohort	Fair	There was no risk factor for short-term Clavien-Dindo complication, including age at surgery [median age 62 days].
Byström et al^26^	2020	30 Hirschsprung patients treated with TAEPT and 30 healthy controls matched for age and gender	22	8	Cross-sectional case–control study	Good	Post-operative BFS (bowel function score) did not show a significant difference in Hirschsprung patients between age groups.[median age at TAEPT was 57 days (12-3,355)]
Bjørnland et al ^27^	2017	200 patients	169	31	Retrospective	Fair	Age at the time of operation (median age 3 months) does not affect the frequency of poor outcomes (stoma, appendicostomy, daily fecal accidents or use of regular enemas).
Yanan Zhang et al^28^	2022	229 neonates	187	42	Cross-sectional	Good	Operation in the neonatal period was quite safe with few complications (age 6-28 days).
Stensrud et al ^31^	2015	52 patients	42	10	Prospective cohort	Good	Internal anal sphincter defects occurred more often in **younger** children(median age 1.8 month)

## Discussion

 The consequences of TAEPT surgery for HD are not always as favorable as the surgeon imagines. Incomplete continence, constipation and postoperative enterocolitis should not be ignored.^33-36^ Previous studies have attempted to investigate the relationship between preoperative characteristics and surgical outcomes in patients with Hirschsprung’s such as age, gender, length of aganglionosis, age at surgery, preoperative enterocolitis, comorbidities, and genetic background.^37-40^ Nevertheless, there is still controversy regarding the best age of operation. Therefore, here, we mainly tried to categorize studies that prefer the neonatal period as the best age against those who believe to postpone it. We mostly focused on 16 studies according to our inclusion and exclusion criteria. In summary, 11 investigations were in favor of postponing the operation to an age above one month, 2 studies found no difference and 3 reported better outcomes in the neonatal period.

 One important issue here is the accuracy of diagnostic modalities for detecting patients. A recent study by Chen et al showed 88.5% correlation between radiological and pathological TZ in rectosigmoid Hirschsprung. This was dependent on the patient’s age. They showed 69% correlation for children under 3 months versus 85.3% (high severity) for older ones.^41^ Overall, the different studies mentioned above indicated that longer periods of disease may lead to better development of radiological TZ. Therefore, determining the transition zone with a high accuracy is necessary. Most children with HD present during the neonatal period with delayed passage of meconium beyond the first 24 hours, abdominal distention, bilious vomiting and feeding intolerance and are diagnosed by a rectal biopsy in the first month of life. However, a definite diagnosis before surgery is mandatory.

 The extent of colon caliber changes depends on the duration of distal bowel obstruction, which is limited in newborns. Therefore, contrast enema is not appropriate in newborns. This is the reason why some surgeons prefer to wait for 1‒2 months. A colon enema performed before the age of 30 days had a sevenfold higher probability of false-negative results.^42^

 The main risk of postponing surgery is the possibility of enterocolitis during the waiting period. This risk can be lowered by ensuring rectal pressure relief with adequate irrigation (usually 10‒20 mL/kg, several times daily), administration of prophylactic metronidazole or probiotics. Due to the risk of enterocolitis, many pediatric surgeons believe that once a diagnosis is made, even in small infants, a laparoscopic or trans-anal operation can be performed successfully and safely.^43^ A survey by the European Society of Pediatric Surgeons found that 33% of pediatric surgeons prefer to perform endorectal pull-through surgery at diagnosis and 67% prefer a delayed approach (4 months or > 5 kg).^44^

 In addition, anorectal manometry is an effective and safe method that complements the diagnosis of HD in newborns. Anorectal sphincter pressure progressively matures with incremental increase during the first months of life ^45-47^.

 Kaiser Decker et al reported that a rectal suction biopsy (RSB) had 81% sensitivity and 97% specificity. Therefore, repeated sampling may be necessary. They found that RSB can also be reliable and safely performed in preterm infants.^48^ However, repeated biopsies in neonates may lead to intestinal perforation. In the study by Putnam et al in clinically suspicious neonates for HD, contrast enema studies showed inconclusive results in 32% of cases.^49^

 Kumar et al concluded that delaying surgery is not logical, and neonates and infants might benefit the most compared to other age groups. As their study design was retrospective with a small number of patients in each sub-group, they could not provide a detailed comparison between neonates and those between 1-12 months. However, the risk of reported complications was higher in neonates.^21^

 Besides, Kastenberg et al compared delayed primary endorectal pull-through ( ≥ 31 days). The median age at operation was 98 days (IQR 61 - 188 days) for patients. They assess 82 patients, 49 neonates and 33 non-neonates. Fifteen neonates compared to five non-neonates developed fecal incontinence (*P* value = 0.13). Besides, enterocolitis and other complications were not different between the two groups. As fecal incontinence was more frequent in neonates, but without a statistically significant difference, the authors tended to conclude that operation in neonates is as safe as those above one month, which is not logical in our opinion. This study had a good methodological design but with a small sample size. Therefore, we might not rely completely on this analysis to advocate surgery in neonates.^22^

 In another investigation, Karlsen et al compared the outcomes of laparoscopic and trans-anal pull-through and reported poorer outcomes in the neonatal period. This study did not fulfill our inclusion criteria but higher complications were reported in neonates.^50^

 In another study by Ivana et al,^32^ no association was found between age at surgery and functional outcomes in Hirschsprung patients. We believe this study lacks a large sample size. Also, they categorized their patients into two groups of those under 4 years and those above, which ignores any classification regarding neonates; therefore, the results might not be very helpful in our data interpretation. Nevertheless, soiling was more frequently reported in patients older than 4 years which is consistent with some other reports. Accordingly, delaying surgery above 2‒4 years is completely erroneous.

 On the other hand, the study by Miyano et al showed that age at surgery was not correlated with postoperative bowel function in HD. However, their emphasis was to validate and highlight their modified laparoscopic technique for HD and their main goal was not comparison between neonates and non-neonates regarding outcomes.^24^

 In addition, Hoff et al reported no risk factor for short-term complication using the Clavien-Dindo grading system, including age at surgery.^25^ They only evaluated outcomes during the first months after surgery as early post-operation complications, and long-term outcomes were notassessed.

 On the other hand, in a multicenter study in Scandinavia to evaluate the predictors of functional outcomes, age at surgery did not have a significant effect on poor outcomes using a multivariate model.^27^ This study was a retrospective investigation mainly to identify long-term complications of children with HDs after operation. Age classification in this study was only given in a table categorized as 0.4 to 1, 1‒2.9, 3‒7.5 and 7.9‒133 months. No detailed information regarding the number of patients in each quartile was found. Their main goal was to assess long-term bowel function and they did not compare complications across different age groups. Therefore, we cannot conclude that the results contradict our recommendation.

 The most reliable study against postponing the operation to above one month was the one conducted by Zhang et al on 229 neonates.^28^ They reported that operation in the neonatal period was quite safe with few complications (age 6‒28 days). This is almost the only well-organized study to defend operation in the neonatal period with a quite large sample size. We admire the authors for the settings they prepared in their study. Nevertheless, we believe that these outcomes are due to the high expertise of the staff in this center as they could recruit 229 patients in 13 years. As most studies reported smaller numbers of patients, we believe delaying the operation to above one month is logical in smaller centers. However, in very few tertiary centers with highly organized settings and experienced pediatric surgeons, we might recommend surgery in neonates.

 In terms of postoperative complications, infants who undergo trans-anal pull-through surgery are exposed to undesirable short-term consequences. Huang et al ^51^ reported that neonates have a longer recovery period after surgery compared to non-neonates. Furthermore, active immunodeficiency and inactive immunity of maternal antibodies result in low resistance to infection in neonates. In a retrospective study evaluating the results of a single-stage trans-anal pull-through in 650 children, the authors concluded that the operation might be more appropriate in the non-neonatal period compared to the neonatal period. Because there was a higher rate of perianal excoriation, anastomotic stenosis and leakage, postoperative enterocolitis and incomplete postoperative continence in neonates than non-neonates.^13^ In addition, Stensrud et al^31^ compared two groups of patients who underwent trans-anal and trans-abdominal surgery regarding anal sphincter damage using ultrasonography. They showed that children who underwent trans-anal pull-through had higher rates of injury. The median age of patients in trans-anal and trans-abdominal surgery was 1.8 (0.4‒133) and 13 (1.2‒100) months. We might conclude that operation at lower ages and using the trans-anal approach would increase the likelihood of sphincter injury.

 A meta-analysis by Westfal et al ^52^ published in 2021 included four studies in addition to their own center’s data to assess the best time to perform the operation for children with HD. They included the findings of Miyano et al,^24^ Zhu et al,^15^ Lu et al^14^ and Chung et al^53^ in their pooled analysis. We discussed the first three above; however, the study by Chung et al^53^ could not be entered because they did not include a clear age classification to separate between neonates and non-neonates. However, as they provided their data sheet, Westfal et al could include it. Overall, Westfal et al claimed that children below 2.5 months of age at surgery would have poorer outcomes, which is somehow in line with the results of our systematic review.

 This study had some limitations. As all systematic reviews, we had to rely on information from other studies. A recall bias is inevitable when compiling information from other investigations. Some studies did not include necessary information needed for our review. Therefore, some high-quality studies might have been excluded due to a high bias score according to the checklist. Besides, some studies included patients with concurrent syndromes which were not assessed in our review. We do not know whether Down syndrome might affect the decision for age selection. We suggest large multicentric studies to collect data on different ethnicities.

## Conclusion

 Despite the recommendation of most studies to treat HD as early as possible, due to more common short-term complications and lower contrast enema diagnostic accuracy in neonates, it seems a reasonable decision to postpone surgery until the child is several months old. However, it is not recommended to delay surgery over 1 year. The overall view on most reviewed articles indicates that age between 3 and 12 months can be a good time for interventional treatment for HD. However, we cannot complain performing surgery in high-volume advanced tertiary centers in the neonatal period.
